# Regulation of Morphology, Aflatoxin Production, and Virulence of *Aspergillus flavus* by the Major Nitrogen Regulatory Gene *areA*

**DOI:** 10.3390/toxins11120718

**Published:** 2019-12-10

**Authors:** Opemipo Esther Fasoyin, Kunlong Yang, Mengguang Qiu, Bin Wang, Sen Wang, Shihua Wang

**Affiliations:** 1 Key Laboratory of Pathogenic Fungi and Mycotoxins of Fujian Province, Key Laboratory of Biopesticide and Chemical Biology of Education Ministry, and School of Life Sciences, Fujian Agriculture and Forestry University, Fuzhou 350002, China; 2018Y90100114@caas.cn (O.E.F.); 1140517012@fafu.edu.cn (M.Q.); 000q120011@fafu.edu.cn (B.W.); 2170517007@fafu.edu.cn (S.W.); 2 Biotechnology Research Institute, Chinese Academy of Agricultural Sciences, Beijing 100081, China; 3 School of Life Science, Jiangsu Normal University, Xuzhou 221116, Jiangsu, China; ykl_long@jsnu.edu.cn

**Keywords:** *Aspergillus flavus*, aflatoxins, nitrogen metabolism, glutamine, AreA

## Abstract

*Aspergillus flavus* is a renowned plant, animal and human pathogen. *areA* is a global nitrogen regulatory gene of the GATA transcription factor family, shown to be the major nitrogen regulator. In this study, we identified *areA* in *A. flavus* and studied its function. The AreA protein contained a signatory zinc finger domain, which is extremely conserved across fungal species. Gene deletion (Δ*areA*) and over-expression (OE::*areA*) strains were constructed by homologous recombination to elucidate the role of *areA* in *A. flavus*. The Δ*areA* strain was unable to efficiently utilize secondary nitrogen sources for growth of *A. flavus*, and it had poorly developed conidiophores, when observed on complete medium, resulting in the production of significantly less conidia than the wild-type strain (WT). Aflatoxin B1 (AFB1) production was reduced in Δ*areA* compared with the WT strain in most conditions tested, and Δ*areA* had impaired virulence in peanut seeds. *areA* also played important roles in the sensitivity of *A. flavus* to osmotic, cell wall and oxidative stresses. Hence, *areA* was found to be important for the growth, aflatoxin production and pathogenicity of *A. flavus*. This work sheds light on the function of *areA* in the regulation of the nitrogen metabolism of *A. flavus,* and consequently aims at providing new ways for controlling the crossover pathogen, *A. flavus*.

## 1. Introduction

*Aspergillus flavus* is a pathogenic soil-borne saprophyte and filamentous fungus which is widely known for its colonization and infection of many important agricultural crops such as cereal grains, tree nuts and legumes in the field, as well as during storage and/or transport [1,2,3,4,5]. *A. flavus* can exploit a wide range of naturally derived nutrient sources, ranging from economic crops and bodies of dead animals to humans and animals with compromised immune systems [5,6,7,8,9], and it produces various secondary metabolites such as the toxic compounds called mycotoxins. The consumption of these compounds is toxic to mammals, with effects ranging from immunosuppression to death in humans [10].

Fungi have the ability to utilize diverse compounds as nitrogen sources. The expression of genes encoding the enzymes and permeases required for nitrogen utilization is regulated by a general mechanism known as nitrogen metabolite repression, which makes them highly expressed in nitrogen-limited and starvation conditions. This allows the preferred use of nitrogen sources that can easily be incorporated as nutrients, such as ammonium and glutamine [11]. Han and colleagues previously reported that glutamine is a preferred nitrogen source in the aflatoxin production of *A. flavus*, with 4 mM limiting threshold concentration [12]. AreA, a highly conserved GATA transcription factor, is the major nitrogen regulatory protein, known for its function of furnishing organisms with the ability to exploit various secondary nitrogen sources. The function of this protein and its homologues have been widely studied in various fungi [13,14,15,16,17,18]. AreA contains a zinc finger domain, with a central loop which plays an important role in the affinity of DNA binding. The L7 (Leucine) residue in the central loop of AreA is reported to be involved in the distinction of recognition elements present in gene promoters [19]. Glutamine and ammonia have been found to inhibit the activity of AreA and NIT2 in *Aspergillus nidulans* and *Neurospora crassa*, respectively, and the presence of excess or free glutamine in the cell triggers NmrA, another protein in the nitrogen metabolism pathway to form a complex with AreA, thereby inhibiting its DNA-binding activity [20,21,22]. In *A. flavus*, the lack of *nmrA* induced a higher transcript level of *areA* in comparison with the wild-type strain [23]. Gln3p, a global regulator in *Saccharomyces cerevisiae*, participating in the expression of diverse genes associated with nitrogen metabolism, was shown to be rapidly dephosphorylated and accumulated in the nucleus as a result of nitrogen starvation and rapamycin addition [24,25]. AreA, its homolog in *A. nidulans*, however, was accumulated in the nucleus only in the absence of preferable nitrogen sources [26,27].

AreA is not only involved in the regulation of nitrogen metabolism, but it also plays certain roles in the secondary metabolite biosynthesis and virulence of pathogens. In *Acremonium chrysogenum,* the deletion of *AcareA* resulted in the loss of the derepression of nitrogen metabolism and decreased the production of cephalosporin [28]. Studies on *Fusarium graminearum* showed that the vegetative growth, nitrogen metabolism, pathogenicity and deoxynivalenol production of the AreA/NIT2 ortholog mutant were significantly affected [29]. NRE, the AreA/NIT2 ortholog in *Penicillium chrysogenum* plays a role in its nitrogen metabolism, and may also regulate penicillin biosynthesis [15,30]. In *Fusarium verticillioides*, fumonisin production is controlled by AREA [31]. Likewise, gibberellin biosynthesis in *Fusarium fujikuroi* is strictly mediated by AREA [14,18,32,33]. The deletion of *areA* in *Colletotrichum gloeospoiodes* results in a significant decrease in vegetative growth and pathogenicity, but increased sporulation [34]. Generally, the major nitrogen regulator, AreA/NIT2, is associated with nitrogen metabolism in a lot of plant pathogenic fungi, but it functions differently and in complicated manners in the pathogenicity of various species. Hence, the need for the investigation of the role of AreA in the plant, animal, and human pathogen *A. flavus*.

In this study, we identified the major nitrogen regulatory gene *areA* in *A. flavus,* encoding a transcription factor made up of 866 amino acids. Although *areA* homologues have been widely studied in fungi, nothing is known of its effect on the morphology, secondary metabolite biosynthesis and virulence of *A. flavus*.

## 2. Results

### 2.1. Identification of AreA from A. flavus

**Figure 1:**
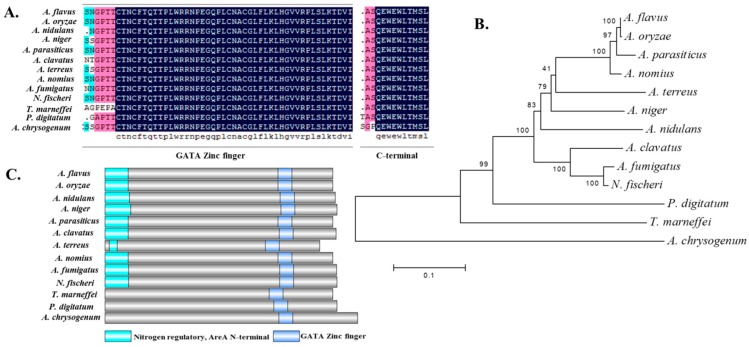
Bioinformatics analysis of major nitrogen regulator AreA. (**A**) Sequence alignment of the AreA protein amino acid sequence from *Aspergillus flavus*, *Aspergillus oryzae*, *Aspergillus nidulans*, *Aspergillus niger*, *Aspergillus parasiticus, Aspergillus clavatus*, *Aspergillus terreus*, *Aspergillus nomius*, *Aspergillus fumigatus*, *Neosartorya fischeri*, *Talaromyces marneffei*, *Penicillium digitatum* and *Acremonium chrysogenum*, showing the conserved domains. (**B**) Phylogenetic tree of the AreA protein from organisms described in panel A. (**C**) Domain prediction of the AreA protein in the aforementioned organisms visualized by DOG 2.0 software.

The AreA protein was identified in *A. flavus* from the FungiDB website, with the sequence ID: AFLA_049870. The amino acid sequences of AreA from *A. flavus* and 12 other fungal species including *Aspergillus oryzae*, *Aspergillus nidulans*, *Aspergillus niger*, *Aspergillus parasiticus*, *Aspergillus clavatus*, *Aspergillus terreus*, *Aspergillus nomius*, *Aspergillus fumigatus*, *Neosartorya fischeri*, *Talaromyces marneffei*, *Acremonium chrysogenum* and *Penicillium digitatum* were obtained from UniProt. The open reading frame (ORF) of the *A. flavus areA* gene consists of 2668 bp, coding for AreA, a transcription factor made up of 866 aa, with a weight of approximately 92.8 KDa. The sequence alignment of the aforementioned organisms revealed that the GATA zinc finger domain and C-terminal of the AreA protein were highly conserved in the aforementioned organisms (Figure 1A). The phylogenetic tree analysis displayed a close evolution between the AreA protein from *A. flavus*, *A. oryzae*, *A. parasiticus*, and *A. nomius*, while the relationship was quite far from the AreA protein of *A. nidulans* (Figure 1B). The protein sequence contains two domains, the Nitrogen Regulatory AreA N-terminal (1–88 aa), and the GATA zinc finger (658–711 aa) (Figure 1C).

### 2.2. Construction of areA Deletion (ΔareA) and Over-Expression (OE::areA) Mutant Strains

**Figure 2:**
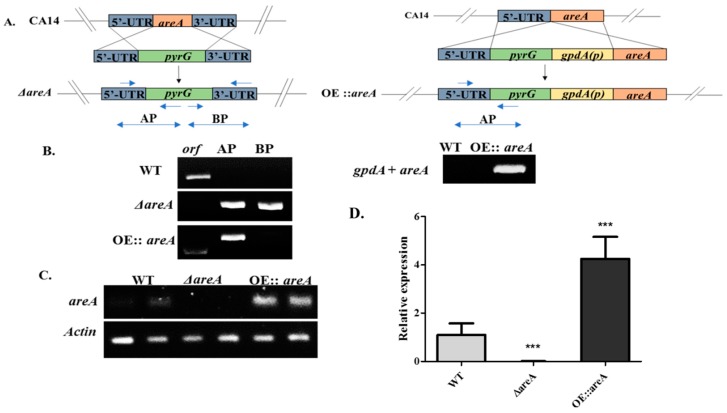
Construction and verification of Δ*areA* and OE::*areA* strains. (**A**) Schematic diagram of the gene manipulation strategy for the construction of Δ*areA* and OE*::areA* strains. (**B**) Diagnostic PCR for the verification of Δ*areA* and OE::*areA* strains. (**C**) RT-PCR verification of WT, Δ*areA* and OE::*areA* strains. (**D**) qRT-PCR verification of WT, Δ*areA* and OE*::areA* strains. Strains were grown on yeast extract–sucrose (YES) medium for 48 h at 37 °C. *** *p* 0.001. Error bars represent the SE (standard error) from three independent experiments with three replicates.

In order to elucidate the role of *areA* in the morphology, secondary metabolite production and pathogenicity of *A. flavus*, the *areA* gene was deleted and over-expressed in *A. flavus* using the homologous recombination method. The obtained transformants were subjected to diagnostic PCR, RT-PCR and qRT-PCR to verify successful gene manipulations. The diagnostic PCR showed the presence of the ORF in the wild-type strain (WT), and the absence of AP (containing part of the 5′ UTR and *pyrG*) and BP (containing part of the 3′ UTR and *pyrG*) fragments, while the Δ*areA* mutant, from which the ORF could not be amplified, displayed the AP and BP amplicons as expected. The diagnostic PCR confirmed the successful deletion of the *areA* gene (Figure 2A). RT-PCR and qRT-PCR analyses showed an undetectable *areA* transcript in Δ*areA*, and a higher *areA* transcript level in OE::*areA* when compared with the WT strain (Figure 2B,C). These results further confirmed the successful construction of the Δ*areA* and OE::*areA* strains.

### 2.3. areA is Important for Nitrogen Utilization and Growth of A. flavus

**Figure 3:**
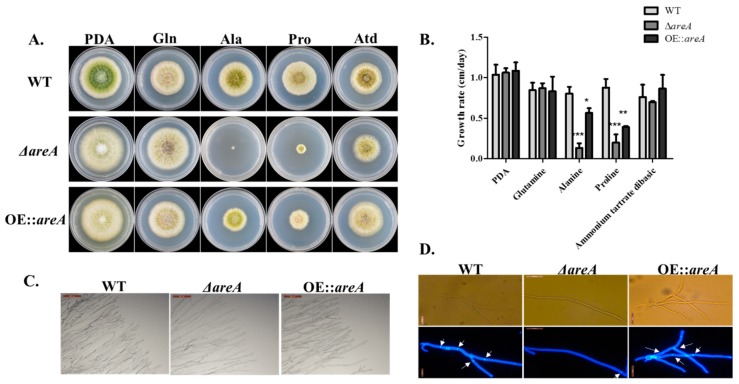
Phenotype, growth rate and mycelial branching of *A. flavus* strains. (**A**) Colony morphology of WT, Δ*areA* and OE::*areA* grown on potato dextrose agar (PDA) and glucose minimal medium (GMM) supplemented with 50 mM Glutamine, alanine, proline, or ammonium tartrate dibasic at 37 °C for 4 d. (**B**) Growth rate analysis of WT, Δ*areA* and OE::*areA* as panel A. (**C**) Mycelial branching of WT, Δ*areA* and OE::*areA* on PDA at 37 °C after 2 d. Bars = 100 µm. (**D**) Septa morphology of WT, Δ*areA* and OE::*areA* grown in PDB at 37 °C overnight. Bars = 20 µm * *p* 0.05, ** *p* 0.01, and *** *p* 0.001. Error bars represent the SE from three independent experiments with three replicates.

The growth of *A. flavus* on solid media was affected by the deletion of *areA*, and aerial hyphae development was inhibited in Δ*areA* on glucose minimal medium (GMM) supplemented with nitrogen sources, with a high severity in Ala and Pro (Figure 3A). The growth assay revealed a slower growth rate of Δ*areA* on glucose minimal media supplemented with Ala and Pro (Figure 3B). The lowest growth rate was observed in GMM + Ala, as the Δ*areA* mutant could barely grow. It was observed that the growth defect of the Δ*areA* mutant could be completely restored on the media supplemented with Gln. Intriguingly, the over-expression strain of *areA* (OE::*areA*) grew poorly on the media supplemented with Ala and Pro compared to the wild type (Figure 3A), which indicated that Ala and Pro may induce the expression of *areA* more largely in the WT strain than in the OE::*areA* strain. Further observation under a microscope revealed that Δ*areA* produced mycelia with a lower density, and fewer branches, in comparison with WT and OE::*areA* strains (Figure 3C). The septa morphology of *A. flavus* strains were also observed, and we discovered that Δ*areA* had significantly fewer septa than the WT and OE::*areA* strains (Figure 3D). These results showed that *areA* is important for the utilization of non-preferred nitrogen sources and growth in *A. flavus*.

### 2.4. areA Influences Conidia Production of A. flavus

**Figure 4:**
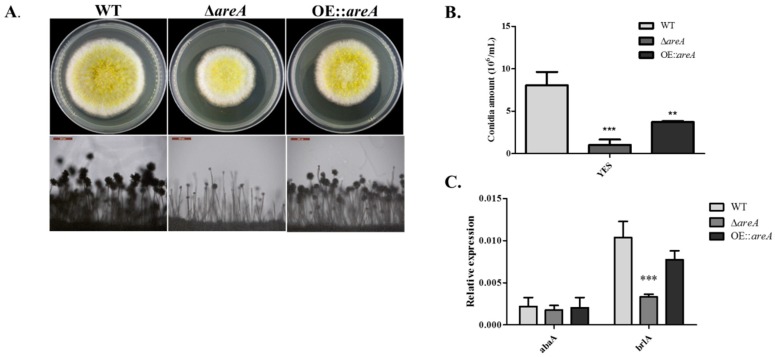
Conidia production of *A. flavus* strains. **(A**) Conidiophore morphology of the WT, Δ*areA* and OE::*areA* strains of *A. flavus* grown on YES medium at 37 °C for 4 d. Bars = 200 µm. (**B**) Amounts of conidia produced by WT, Δ*areA* and OE::*areA* strains grown on YES medium at 37 °C for 4 d. (**C**) The relative expression levels of the conidiation-related genes *abaA* and *brlA* in the WT, Δ*areA* and OE::*areA* strains of *A. flavus*. ** *p* 0.01 and *** *p* 0.001. Error bars represent the SE from three independent experiments with three replicates.

The conidiophore morphology of the *A. flavus* strains was observed using a microscope, and we observed that the Δ*areA* mutant was severely impaired in the formation of conidiophores and failed to form visible conidia due to the undeveloped phialides (Figure 4A). Further, the amount of conidia produced by Δ*areA* on yeast extract–sucrose (YES) medium was significantly lower than those of the WT and OE::*areA* strains (Figure 4B). To further understand the results obtained, the transcript levels of the conidia-related genes, *abaA* and *brlA*, were assessed, and we discovered that the transcript level of *brlA* was significantly reduced in Δ*areA* compared to WT and OE::*areA* strains (Figure 4C). These results indicated that *areA* is required for the full conidiation of *A. flavus*.

### 2.5. areA Impedes Sclerotia Formation in A. flavus

**Figure 5:**
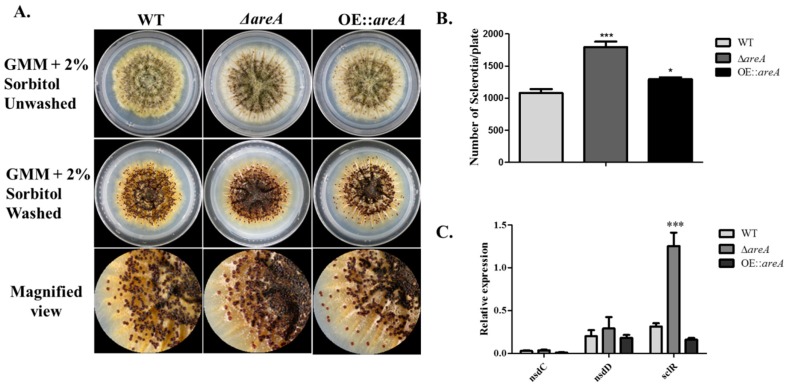
Sclerotia production of *A. flavus* strains. (**A**) Sclerotia production (before and after washing off the conidia) of the WT, Δ*areA* and OE::*areA* strains on glucose minimal medium (GMM) at 37 °C. (**B**) Statistical analysis of the sclerotia production. (**C**) The expression levels of *nsdC*, *nsdD* and *sclR* genes involved in sclerotia production by qRT-PCR assay. * *p* 0.05 and *** *p* 0.001. Error bars represent the SE from three independent experiments with three replicates.

The amount of sclerotia produced by the *A. flavus* strains was assessed by growing the strains on GMM supplemented with 2% sorbitol, with Gln as the sole nitrogen source. It was discovered that more sclerotia were produced by Δ*areA* in comparison with the WT and OE::*areA* strains (Figure 5A,B). To shed more light on the result obtained, the transcript levels of sclerotia-related genes, *nsdC*, *nsdD* and *sclR*, were examined, and we found that the transcript levels of *nsdC* and *nsdD* genes were similar in all the three test strains, while *sclR* was significantly increased in Δ*areA* compared to the WT and OE::*areA* strains (Figure 5C). These results suggested that *areA*, being a nutrition gene, was important for the assimilation of nutrients by *A. flavus*.

### 2.6. areA Influences the Stress Responses of A. flavus

**Figure 6:**
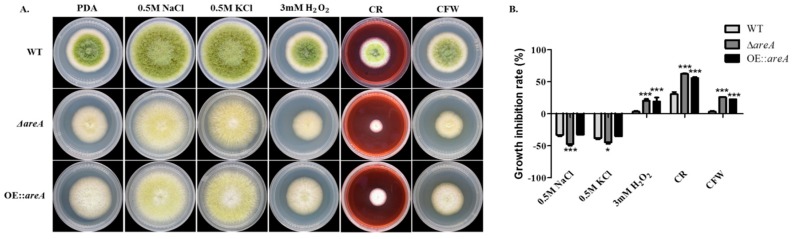
Inhibition of the growth rate of *A. flavus* strains under different stress conditions. (**A**) Colony morphology of the WT, Δ*areA* and OE::*areA* strains grown on PDA, or PDA containing 0.5 mol/L NaCl and KCl, 3 mM H_2_O_2_, 300 μg/mL Congo Red (CR), and 100 μg/mL calcofluor white (CFW) at 37 °C for 4 d, respectively. (**B**) The inhibition of the growth rate of the strains in panel A. * *p* 0.05 and *** *p* 0.001.

The effect of *areA* deletion on the response of *A. flavus* to osmotic stress was examined by culturing the strains on potato dextrose agar (PDA), supplemented with NaCl and KCl (Figure 6A), and we observed that osmotic stress enhanced the growth of *A. flavus* (Figure 6B). In the oxidative stress assay, both the Δ*areA* and OE::*areA* strains displayed significant growth inhibition in the presence of H_2_O_2_ compared to the wild type (Figure 6). Cell wall stress was induced by two stress agents, Congo Red (CR) and calcofluor white (CFW) (Figure 6A), and the result showed that the relative growth rate of the Δ*areA* and OE::*areA* strains were significantly inhibited in comparison to the wild-type strain (Figure 6B). These results suggested that *areA* may play a role in the sensitivity of *A. flavus* to osmotic, oxidative and cell wall stresses.

### 2.7. Aflatoxin Biosynthesis is Partially Regulated by AreA

**Figure 7:**
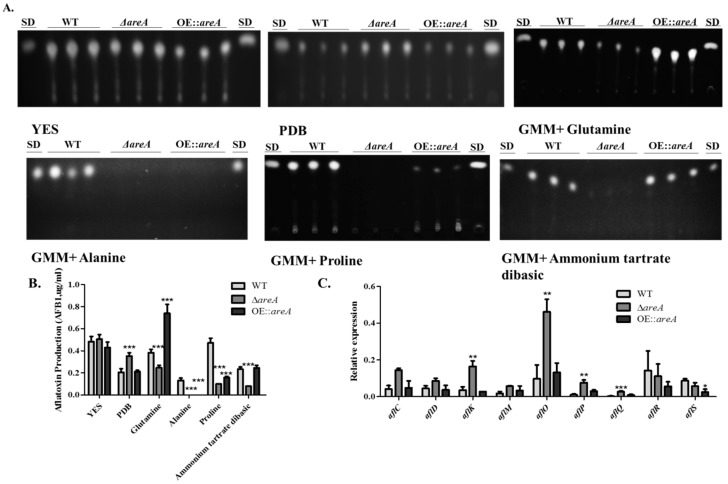
Aflatoxin B1 (AFB1) biosynthesis of *A. flavus* strains. (**A**) Thin layer chromatography (TLC) assay of AFB1 produced by WT, Δ*areA* and OE::*areA* strains grown on YES medium, PDB and GMM supplemented with 50 mM Gln, Pro, Ala or NH_4_ at 29 °C for 6 d. (SD indicates standard AFB1.) (**B**) Quantification assay of AFB1 produced in panel A. (**C**) The expression levels of *aflC*, *aflD*, *aflK*, *aflM*, *aflO*, *aflP*, *aflQ*, *aflR*, and *aflS* genes involved in aflatoxin biosynthesis by qRT-PCR assay. Error bars represent the SE from three independent experiments with three replicates. * *p* 0.05, ** *p* 0.01 and *** *p* 0.001.

Aflatoxin B1 (AFB1) is the most important and toxic secondary metabolite produced by *A. flavus*. Here, we found by thin layer chromatography (TLC) assay, that the lack of *areA* promoted AFB1 biosynthesis in PDB medium compared to the wild-type and over-expression (OE::*areA*) strain (Figure 7A). However, there was no significant difference observed in the AFB1 production of the three strains in YES medium. Intriguingly, when glutamine or ammonium tartrate dibasic was used as the sole nitrogen source, AFB1 production was inhibited in Δ*areA*, but accumulated in OE::*areA* (Figure 7A,B). As expected, the Δ*areA* and OE::*areA* strains produced decreased amounts of AFB1 in the presence of proline and alanine, since these two strains grew poorly when proline and alanine were used as the sole nitrogen source. Although the activity of AreA might be inhibited in the wild-type strain in the presence of glutamine or ammonium, the wild-type strain produced detectable AFB1 (Figure 7A,B). We investigated the expression levels of some genes in the aflatoxin biosynthesis gene cluster (BGC), and we discovered that the expression levels of the cluster activator, *aflR*, and enhancer, *aflS* were not significantly reduced in the Δ*areA* strain when compared with the WT strain, while the expression levels of the other genes examined (*aflK*, *aflO*, *aflP* and *aflQ*) were significantly increased (Figure 7C). These results suggested that AFB1 biosynthesis in *A. flavus* was partially regulated by *areA* and may be influenced via a different pathway independent of AreA from nitrogen metabolism.

### 2.8. areA is Necessary for the Pathogenicity of A. flavus

**Figure 8:**
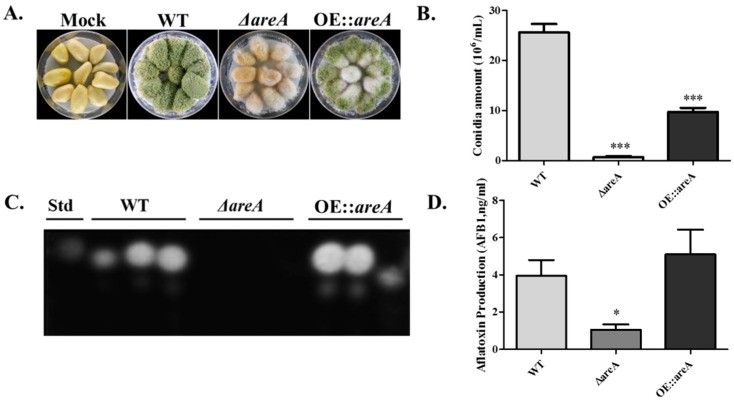
Pathogenicity assay of *A. flavus*. (**A**) Morphology of *A. flavus* WT, Δ*areA* and OE::*areA* strains on peanut seeds after 6 d of inoculation. (**B**) Conidia production of strains in panel A. (**C**) TLC analysis of AFB1 extracted from panel A. (SD indicates standard AFB1.) (**D**) Quantification of TLC result in panel C. * *p*0.05 and *** *p*0.001. Error bars represent the SE from three independent experiments with three replicates.

*A. flavus* is known to readily colonize oil-rich crop seeds. Hence, we investigated the effect of *areA* deletion on the colonization of peanut seeds. The assay revealed that the loss of *areA* caused a significant impairment in the pathogenicity of the strain on peanut seeds (Figure 8A). The pathogenicity of the strains was assessed based on the mycelia and conidia produced on the surface of the infected seeds. The result showed that the Δ*areA* mutant grew less vigorously on peanut seeds (Figure 8A). Further, the conidia quantification assay revealed that Δ*areA* was significantly impaired in conidiation compared to the WT and OE::*areA* strains (Figure 8B). Further quantification of AFB1 production from the infected plant seeds showed a significant decrease in Δ*areA* in comparison with the WT strain (Figure 8C,D). These results suggested that *areA* is necessary for the pathogenicity of *A. flavus.*

### 2.9. Subcellular Localization of AreA in A. flavus

**Figure 9:**
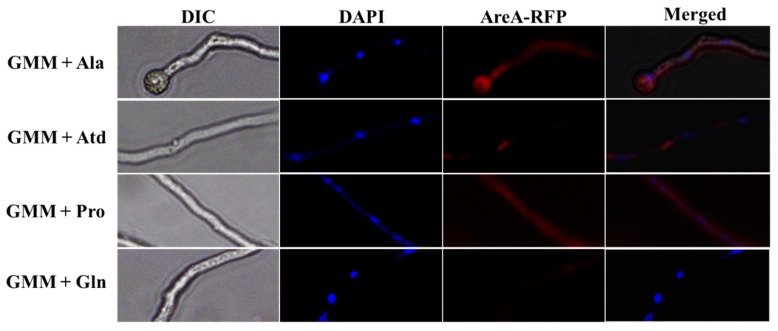
Subcellular localization of AreA in *A. flavus*. Confocal scanning images of AreA::RFP in vegetative mycelium. The AreA::RFP strain was cultured for 16 h at 37 °C in GMM supplemented with 50 mM Gln, Pro, Ala or NH_4_. Bars = 10 µm.

The subcellular localization of AreA was investigated by culturing *A. flavus* strains expressing areA tagged with RFP (red fluorescence protein) in GMM supplemented with different nitrogen sources. The samples were stained with 4,6-diamidino-2-phenylindole (DAPI) to enable a clear view of the nucleus. AreA was seen to be localized both in the nucleus and in the cytoplasm, under a nitrogen-limited condition (presence of alanine and proline), mainly in the cytoplasm in the presence of ammonium, while the signal could barely be seen under a nitrogen-repressed condition (presence of glutamine) (Figure 9). This result confirms the transcription activity of AreA and its involvement in nitrogen metabolism.

## 3. Discussion

The ability of *A. flavus* to utilize a wide range of nutrients with different qualities and quantities is essential for its pathogenicity, as it has previously been shown that the expression of virulence-related genes is induced by nitrogen starvation [18,20]. Fungi are able to utilize several nitrogen sources, subject to the regulatory mechanism NMR, which allows the use of a preferred nitrogen source like glutamine and ammonium over secondary nitrogen sources [32,35]. In this study, we characterized the function of the major nitrogen regulatory gene *areA* in *A. flavus*. Its deletion resulted in a defective utilization of secondary nitrogen sources, and a slightly ineffective use of ammonium, which is a preferred source of nitrogen in *A. nidulans* [36]. In consonance with our findings, the ineffective use of ammonium has been recorded in the deletion of an *areA* ortholog in *A. oryzae* [37] and *F. graminearum* [35]. Here, we found that the absence of *areA* was only compensated for, by the presence of glutamine, in the utilization of nutrients for proper growth, suggesting that glutamine is a preferred source of nitrogen for *A. flavus*. This is in contrast with the study of *areA* in *A. nidulans*, where glutamine is a non-preferred source [36]. A study performed by Min and colleagues also showed that neither glutamine nor ammonium is a preferred nitrogen source in *Fusarium zeae*, but rather urea [38].

*areA*, being the major regulator of nitrogen metabolism, is expected to affect the vegetative development of *A. flavus*, as nitrogen is among the most essential nutrients for the growth and differentiation of organisms [39]. Hence, we investigated this by examining the colony diameter of *A. flavus* strains on different culture media, and also by viewing the mycelial branching and septa morphology of *A. flavus* strains grown in complete medium. The degree of branching of fungal mycelial is essential for the assimilation of nutrients by the fungus, and the presence of septa indicates the growth and maturation of new cells. We discovered that *areA* deletion led the formation of less dense mycelial branches and few septa, indicating that *areA* positively regulates the growth and development of *A. flavus*.

The regulation of conidiation in filamentous fungi involves certain regulators such as *VeA/Ve1*, *VelB*, *WetA*, *brlA* and *abaA* [40]. In the fruit postharvest pathogen *Colletotrichum gloeosporioides*, the deletion of *CgareA* up-regulated *Ve1*, resulting in an increased conidia production [34]. Here, we found that *areA* influenced the conidiation of *A. flavus*, as its deletion resulted in a significantly decreased the amount of conidia when grown on both complete and minimal media containing different nitrogen sources. Further, we found that the conidiophore produced by the Δ*areA* mutant was poorly formed, which is consistent with the down-regulated the expression level of *brlA* observed in the *areA* deletion strain. These data suggested that the absence of *areA*, not the quality of nitrogen source, was the cause of the reduction of conidia production in *A. flavus*.

The production of secondary metabolites in fungi is influenced by the available nitrogen sources and nitrogen regulators [39,41,42]. GATA transcription factors are known to influence the utilization of nutrients, morphology or growth of *Aspergillus*, and their disruption may also cause a significant down-regulation of the AF biosynthesis genes expression, but not a total lack [43]. AreA is a positive regulator of the expression of genes related to the production of several secondary metabolites like GA, fumonisin, DON, zearalenone, fusarielin H, beauvericin and cephalosporin [43]. AreA, as a GATA transcription factor, has binding sites in the promoters of key genes in the AF biosynthesis cluster [44], suggesting that AreA may have a direct influence on the expression of these genes. The *aflJ*-*aflR* (*aflJ*, now called *aflS*) intergenic region also has approximately five AreA binding sites [43]. Additionally, certain aflatoxigenic strains of *A. flavus*, *A. sojae*, and *A. oryzae* reported to have full transcription of *aflR*, but had no expression of *aflO*, produced no aflatoxin. This implies that although *aflR* may induce the transcription of AF biosynthesis pathway genes, other factors may affect the expression levels of the genes [45]. We investigated the expression levels of some genes in the AF BGC in *A. flavus* when cultured on YES medium, including the cluster activator, *aflR*, the cluster expression enhancer, *aflS*, and some other genes responsible for the production of the AF pathway intermediates (*aflC*, *aflD*, *aflK*, *aflM*, *aflO*, *aflP*, and *aflQ*). We discovered that the expression levels of *aflR* and *aflS* were not significantly down-regulated in Δ*areA* in comparison with the WT strain, however, the expression levels of *aflK*, *aflO*, *aflP* and *aflQ* were significantly up-regulated in the Δ*areA* strain. Our results in conjunction with previous findings suggested that in *A. flavus*, although *aflR* is responsible for the activation of the transcription of the AF BGC, additional factors may affect the expression levels of the pathway genes. It has been previously reported that, the nitrogen sources available to *A. flavus* affected its biosynthesis of AFB1, and glutamine was reported by a previous study in our lab as the optimal nitrogen source for the production of AFB1 [12]. However, in this study, we discovered that the WT strain produced more AFB1 in GMM supplemented with proline as the nitrogen source than in the presence of glutamine, while the highest amount of AFB1 was produced by OE::*areA* when grown in GMM supplemented with glutamine. The gene deletion mutant grows better than the WT strain on glutamine. This suggests that as much as glutamine inhibits the function of *areA*, the presence of *areA* does not give room for the complete utilization of glutamine as a nitrogen source. However, in the case of proline, the gene deletion mutant grew poorly, which indicated that the utilization of proline needs *areA*. This implied that the WT strain may be able to utilize proline better than glutamine, and this was evident in the slightly increased colony size and AFB1 quantity in the proline-containing medium. On the other hand, despite the ability of proline to be utilized as both nitrogen and carbon source, it could not rescue the lack of the function of *areA* in AFB1 production, and this may be due to carbon catabolite repression induced by glucose [46]. It has been previously shown in *A. nidulans* that AreA is not sufficient for the utilization of proline, and the presence of CreA hinders the action of the element required for its full utilization [47]. The loss of *areA* and the presence of *creA* therefore poses a double-fold hindrance to the utilization of proline, and this could be the cause of the inability of the *areA* deletion mutant of *A. flavus* to utilize proline both for growth and AFB1 production.

The system responsible for the regulation of osmotic stress in fungi has previously been shown to be connected to fungal development, and previous studies show that in *A. flavus*, osmotic stress induced by high concentrations of NaCl, sorbitol, and KCl has positive effects on vegetative growth, leading to increased conidiation [48]. In this study, we observed that osmotic stress induced by NaCl and KCl not only improved the growth of the wild-type strain of *A. flavus*, but that of the Δ*areA* mutant was also significantly increased. This may be as a result of the optimal utilization of the materials and energy dispensed by the fungus for development in a bid to provide favorable survival conditions, in response to osmotic stress [48], by the Δ*areA* mutant. Due to the ability of the Δ*areA* mutant to conveniently utilize whichever nutrient source available to it, as it does not have to strive for the utilization of non-preferred nitrogen sources, according to the function of the *areA* gene.

During the colonization of hosts by pathogenic fungi, the ability to surmount diverse detrimental environmental conditions, especially an oxidative surge which may lead to an accumulation of extremely harmful reactive oxygen species (ROS), is a requirement for fungal pathogens. Plants have been shown to produce harmful ROS, as a form of defense to counter pathogens [49,50]. Because of the relative stability of H_2_O_2_ and its ability to easily diffuse through membranes, it acts as a means of communication for cells to initiate defense response [51]. H_2_O_2_ is also synthesized in large amounts in a mechanism known as a hypersensitive reaction (HR), employed by plant cells to counter the invasion of pathogens [52,53]. Here, we observed that the growth of the Δ*areA* mutant was significantly inhibited by oxidative stress, which implied that the gene *areA* may play a role in reducing the susceptibility of *A. flavus* to oxidative stress, which further implies that *areA* may help shield *A. flavus* from the counter attacks of the host plant during infection.

Snoeijers and colleagues showed that the accessibility of nitrogen is important for colonization and pathogenicity [54]. The virulence of *A. flavus* has been said to be dependent on several factors, one of which is not aflatoxin [6]—unlike in *F. zeae* wheat head blight, where trichothecenes are virulence factors [55]. It has been reported that a shortage in the nitrogen supply of plant pathogens at the start of the infection process gives a signal for the commencement of infection [25,56]. In this study, the ability of *A. flavus* to effectively colonize hosts was impaired by the deletion of *areA*, and the conidiation and AFB1 production of the Δ*areA* mutant were found significantly reduced on hosts. These results were in consonance with the AreA studies in *F. verticillioides* [31], *Ustilago maydis* [56], and *C. gloeosporioides* [34], but different from that of *Magnaporthe grisea* [16].

Transcription factors are localized to the nucleus under conditions in which they carry out their transcription activity [57]. Hence, it is expected that AreA would be localized in the nucleus under nitrogen starvation conditions, as it effects the expression of genes related to the exploitation of less preferred nitrogen sources. We discovered that AreA was localized in the nucleus and also in the cytoplasm under nitrogen starvation conditions, while it was mainly localized in the cytoplasm in the presence of ammonium. 

In conclusion, AreA, as a global transcription factor, is involved in many pathways and mechanisms in *A. flavus* other than nitrogen metabolism. Further, the gene is important for both the primary and secondary metabolism of *A. flavus*, irrespective of the nitrogen source present. However, further studies need to be carried out to elucidate the mechanism through which *areA* plays its roles in *A. flavus*.

## 4. Materials and Methods

### 4.1. Strains and Culture Conditions

The fungal strains and plasmids used in this study are listed in Table 1. In this study, the culture media used include, glucose minimal medium (GMM, 10 g/L glucose, 6 g/L NaNO_3_, 1.52 g/L KH_2_PO_4_, 0.52 g/L KCl, 0.52 g/L Mg_2_SO_4_∙7H_2_O, and 1 mL trace elements), yeast extract–sucrose (YES, 20 g/L yeast extract, 150 g/L sucrose, 1 g/L Mg_2_SO_4_∙7H_2_O), yeast extract–glucose agar (YGT, 5 g/L yeast extract, 20 g/L glucose, 1 mL trace elements) with or without uracil and uridine, potato dextrose agar (PDA, BD Difco™, Franklin, NJ, USA), potato dextrose broth (PDB, BD Difco™, Franklin, NJ, USA) and Czapek agar (CA, BD Difco™, Franklin, NJ, USA, 1 M sucrose, 10 mM ammonium tartrate dibasic). 15 g/L agar was added for solid media. All strains were cultured at 37 °C for growth, and 29 °C for aflatoxin analysis [23,58,59]. All experiments were carried out in triplicate, with each strain having four plates.

### 4.2. Bioinformatics Analysis of AreA Sequence

The AreA protein sequences of *A. flavus* (AFLA_049870) were obtained from FungiDB, and *A. oryzae* (O13415), *A. nidulans* (P17429), *A. fumigatus* (A0A0J5PGE9), *A. parasiticus* (Q9Y7E8), *A. clavatus* (A1CMX8), *A. niger* (O13412), *A. nomius* (A0A0L1IRC7), *A. terreus* (Q0CGC8), *Neosartorya fischeri* (A1DL08), *Penicillium digitatum* (K9G1P2), *Talaromyces marneffei* (A0A093VHJ1), and *Acremonium chrysogenum* (S5YAT5) were obtained from UniProt (www.uniprot.org). The phylogenetic tree was constructed with the downloaded sequences, using MEGA 5.1 software [61]. Domain prediction of the AreA protein in the aforementioned organisms was visualized by DOG 2.0 software (Lab of Cell Dynamics, and Lab of Nanobiology, University of Science & Technology of China, Hefei, Anhui, China, 2014).

### 4.3. Targeted Deletion and Over-Expression of the A. flavus areA Gene

We created the *areA* gene deletion mutant (Δ*areA*) using the method of homologous recombination, in which the ORF of *A. flavus areA* was replaced by *A. fumigatus pyrG*. We constructed a vector A-*pyrG*-B, containing 1000 bp of the sequences flanking the *areA* gene, both upstream and downstream, and *pyrG*. These three fragments were fused by overlap PCR. A and B were amplified from the genomic DNA of *A. flavus* using the primer pairs *areA*-AF/*areA*-AR and *areA*-BF/*areA*-BR, respectively, and Af*pyrG* gene was amplified with the primers *pyrG*-F/*pyrG*-R. The overlap PCR was carried out using a pair of nested primers *areA*-NF/*areA*-NR. The resulting construct was then transformed into the protoplasts of *A. flavus* SRRC1709 [62]. The over-expression strain (OE::*areA*) was constructed by replacing the native promoter of *A. flavus areA* with another promoter from *A. nidulans*, *gpdA*(p). This was also carried out by homologous recombination, and the vector A-*pyrG*-*gpdA*(p)-*areA,* containing A and *pyrG* as in the deletion mutant, was constructed. The *gpdA*(p) fragment was amplified from the gDNA of *A. nidulans* with the primer pair *gpdA*(p)-F/*gpdA*(p)-R, and *areA* was amplified by the primer pair A-*gpdA*-F/A-*gpdA*-R. The fragments were fused together by overlap PCR with the primers A-*gpdA*-NF and A-*gpdA*-NR. The resulting construct was then transformed into the *A. flavus* SRRC1709 strain. Gene-specific primers are shown in Table 2.

### 4.4. Growth, Conidia and Sclerotia Production Analysis

PDA and GMM supplemented with 50 mM nitrogen source (glutamine, Gln; alanine, Ala; proline, Pro; or ammonium tartrate dibasic, NH_4_) agar media were used for analysis of growth rate. A total amount of 10^6^ conidia of each strain (WT, Δ*areA*, and OE::*areA*) were point-inoculated onto plates containing the aforementioned media. Each medium had 4 replicates. The plates were incubated at 37 °C for 4 d in the dark, with a daily measurement of the colony diameter of each strain on every plate. Mycelial branching of *A. flavus* strains was observed by point inoculating the strains on a glass slide covered with a thin layer of PDA medium and culturing at 37 °C for 2 d. Septa morphology was observed from an overnight culture of *A. flavus* strains at 37 °C, and the culture was stained with CFW to enable a clear view of the diaphragm.

*A. flavus* strains were cultured on YES medium at 37 °C for 4 d. Three plugs were taken along a radius of each plate into new tubes after 4 d, then conidia were homogenized and diluted with 5 mL distilled water and counted using a hemocytometer under a microscope. Conidiophores were observed from 2-day-old cultures of *A. flavus* strains on YES medium, cultured at 37 °C for 2 d in the dark. After 2 d, the plates were collected, and the spores and hyphae were scraped off the surface of the medium, making the mycelia visible. Mycelia were cut into short strips and placed on glass slides, which were in plates lined with moistened filter paper. The plates were then incubated at 37 °C for 12 h under light conditions. The mycelia strips were viewed under a microscope to observe conidiophore formed. Sclerotia production was analyzed using a method previously described with a slight modification [4,59]. Concisely, 10^6^ spores of each strain were point-inoculated on GMM supplemented with 2% sorbitol and 10 mM Gln as the nitrogen source and cultured at 37 °C for 8 d in dark conditions. After 8 d, the plates were sprayed with 75% ethanol to wash away conidia and expose sclerotia. The sclerotia produced on each plate were then counted.

### 4.5. AF extraction and Analysis

AF extraction was carried out from *A. flavus* liquid cultures in YES, PDB, and GMM supplemented with 50 mM nitrogen sources, and analyzed using a previously described TLC (thin layer chromatography) method [63].

### 4.6. Stress Assay

PDA was supplemented with different stress agents, NaCl (0.5 M) and KCl (0.5 M) for osmotic stress, H_2_O_2_ (3 mM) for oxidative stress, Congo Red (CR, 0.3 mg/mL), calcofluor white (CFW, 0.1 mg/mL) and SDS (0.1 mg/mL) for cell wall stress. 10^6^ conidia of *A. flavus* was point-inoculated onto the various media and incubated at 37 °C for 4 d in dark condition. Each strain had three repeats for every type of stress, and the experiments were repeated at least thrice [58].

### 4.7. Pathogenicity Assay

Peanut seeds were used to analyze the pathogenicity of the *A. flavus* strains by previously described methods [4,64,65]. The amount of conidia and aflatoxin production were analyzed using the same methods described above.

### 4.8. Subcellular Localization

The localization strain AreA::RFP, in which *areA* was tagged with RFP, was constructed using the homologous recombination method. The *RFP* gene was tagged to the end of the *areA* gene, just before its stop codon, so that *RFP* would be expressed alongside *areA*. A vector containing the *areA* coding region, *RFP*, *pyrG*, and the 3′ flanking sequence of *areA* was constructed and transformed into SRRC1709 protoplasts. The fragments were amplified using the following primer pairs: AR-F/AR-R, RFP-F/RFP-R, *pyrG*-F/*pyrG*-R, and *areA*-BF/*areA*-BR for *areA* coding region, RFP, *pyrG*, and the 3′ flanking sequence of *areA*, respectively. The AreA::RFP strain was cultured in 1.5 mL EP tubes containing 500 µL GMM, supplemented with different nitrogen sources, 50 mM (Gln, Ala, Pro, and NH_4_), at 37 °C for 16 h, in a shaker. The medium was discarded from the tubes, and hyphae were crushed and washed with phosphate buffer saline (PBS) at least 3 times. Then, 1 mg/mL 4,6-diamidino-2-phenylindole (DAPI) was added to the tubes now containing PBS, and incubated on ice for 15 min, away from light. Hyphae were picked onto glass slides and viewed under a confocal microscope.

### 4.9. Quantitative Reverse Transcription Polymerase Chain Reaction

Mycelia were harvested at 72 h post-inoculation on YES medium from all strains. Total RNA was extracted using TRIzol reagent (Biomarker Technologies, Beijing, China), and the first strand cDNA was synthesized using *TransScript^®^* One-Step gDNA Removal and cDNA Synthesis SuperMix (Transgen Biotech, Beijing, China). qRT-PCR was performed using the Pikoreal 96 Real-time PCR System (ThermoFisher Scientific, Waltham, MA, USA), with Pikoreal™ 2.2 software (ThermoFisher Scientific, Waltham, MA, USA) and SYBR Green supermix (Takara, Beijing, China). The qRT-PCR conditions were as follows: 95 °C for 7 min, 40 cycles of 95 °C for 5 s, and 60 °C for 30 s. The 2^−^^ΔΔCT^ method was used to calculate relative expression of transcripts [66]. The Ct values for *actin* and *areA* were obtained from all *A. flavus* strains, with WT as the control. The Ct values of actin obtained from the *A. flavus* strains were then subtracted from the Ct values of *areA* in the respective strains. The value of ∆∆CT was calculated from the resulting differences, and 2^−^^ΔΔCT^ was then used to obtain the expression fold change. *Actin* was used as an internal control for the normalization of the expression data. All qRT-PCR primers were listed in Table 2.

### 4.10. Statistical Analysis

Data are presented as the mean ± standard deviation (SD) of three biological replicate samples. Statistical and significance analysis were carried out using GraphPad Prism 5, and significance was recognized if *p*-values were 0.05. All results from the assays were differentiated by comparing the mutant strains (Δ*areA* and OE::*areA*) to the wild-type strain (WT) using one-way analysis of variance.

## Figures and Tables

**Table 1:** Strains used in this study.

Strain	Characterization	Source
*A. flavus SRRC1709* (CA14PTS)	∆*ku70*, ∆*pyrG*, and ∆*niaD,* Used for gene deletion	[60]
WT	∆*ku70*, ∆*pyrG*⸬Af*pyrG*, ∆*niaD*	This study
∆*areA*	∆*ku70*, ∆*pyrG*⸬Af*pyrG*, ∆*niaD,* ∆*areA*	This study
OE::*areA*	∆*ku70*, ∆*pyrG*, ∆*niaD, gpdA*(*p*)⸬*areA*⸬Af*pyrG*	This study

**Table 2:** Primers used in this study.

Primer Name	Sequence 5′ to 3’	Application
*areA*-F	ATTCGTAATACCTGCGTTCC	*areA* gene cloning
*areA*-R	GGGTGAAGAGCATTGTTTGAGGCCAGTCTACCCGCCCTAAA	
*areA*-AF	ATTCGTAATACCTGCGTTCC	5′ UTR fragment amplification
*areA*-AR	GGGTGAAGAGCATTGTTTGAGGCCAGTCTACCCGCCCTAAA	
*areA*-BF	GCATCAGTGCCTCCTCTCAGACGAGGTGCAATGCGTTGGT	3′ UTR fragment amplification
*areA*-BR	CTGGCCTGAAAGTGGGTG	
*pyrG-*F	GCCTCAAACAATGCTCTTCACCC	*pyrG* amplification
*pyrG-*R	GTCTGAGAGGAGGCACTGATGC	
*areA*-OF	CCCAGTTGCCCAACCAGGAG	*areA* ORF verification
*areA*-OR	GGTCGAGTAATTGGTGGCGTTC	
*areA-*NF	GTTTGACCGTCGCCTCAGTA	Fusion PCR
*areA-*NR	GGGTGGGTTGTTCGTGTTAG	
*A-gpdA*-F	CTTTCCCACTTCATCGCAGCTTGATGTCCGGGTTAACCCTCGG	*areA* ORF amplification for over-expression
*A-gpdA*-R	GGGCGTCCAAGGCATAATCG	
*gpdA*-F	GATCCCGTAATCAATTGCCCCATCCGGATGTCGAAGGCTT	*gpdA*(p) amplification
*gpdA*-R	GTGATGTCTGCTCAAGCGGGG	
P801-R	CAGGAGTTCTCGGGTTGTCG	AP fragment verification
P1020-F	ATCGGCAATACCGTCCAGAAGC	BP fragment verification
*abaA*-F	TCTTCGGTTGATGGATGATTTC	qRT-PCR qRT-PCR
*abaA*-R	CCGTTGGGAGGCTGGGT	
*brlA*-F	GCCTCCAGCGTCAACCTTC	qRT-PCR qRT-PCR
*brlA*-R	TCTCTTCAAATGCTCTTGCCTC	
*sclR*-F	CAATGAGCCTATGGGAGTGG	qRT-PCR qRT-PCR
*sclR*-R	ATCTTCGCCCGAGTGGTT	
*nsdC*-F	GCCAGACTTGCCAATCAC	qRT-PCR qRT-PCR
*nsdC*-R	CATCCACCTTGCCCTTTA	
*nsdD*-F	GGACTTGCGGGTCGTGCTA	qRT-PCR qRT-PCR
*nsdD*-R	AGAACGCTGGGTCTGGTGC	
*areA*-F	GAAACGGACGAGGCTAACAA	qRT-PCR qRT-PCR
*areA*-R	ATACTATGGTTCGCCGGATTG	
*aflO*-F	GATTGGGATGTGGTCATGCGATT	qRT-PCR
*aflO*-R	GCCTGGGTCCGAAGAATGC	
*aflQ*-F	GTCGCATATGCCCCGGTCGG	qRT-PCR
*aflQ*-R	GGCAACCAGTCGGGTTCCGG	
*aflC*-F	GTGGTGGTTGCCAATGCG	qRT-PCR
*aflC*-R	CTGAAACAGTAGGACGGGAGC	
*aflD*-F	GTGGTGGTTGCCAATGCG	qRT-PCR
*aflD*-R	CTGAAACAGTAGGACGGGAGC	
*aflM*-F	ATGTCCGACAACCACCGTTTAGATGGCA	qRT-PCR
*aflM*-R	CAATGATCTTTCCACTTACCCATTCGGCTG	
*aflK*-F	GAGCGACAGGAGTAACCGTAAG	qRT-PCR
*aflK*-R	CCGATTCCAGACACCATTAGCA	
*aflP*-F	ACGAAGCCACTGGTAGAGGAGATG	qRT-PCR
*aflP*-R	GTGAATGACGGCAGGCAGGT	
*aflR*-F	AAAGCACCCTGTCTTCCCTAAC	qRT-PCR
*aflR*-R	GAAGAGGTGGGTCAGTGTTTGTAG	
*actin*-F	ACGGTGTCGTCACAAACTGG	qRT-PCR qRT-PCR
*actin*-R	CGGTTGGACTTAGGGTTGATAG	
